# Intratumoral localization and activity of 17β-hydroxysteroid dehydrogenase type 1 in non-small cell lung cancer: a potent prognostic factor

**DOI:** 10.1186/1479-5876-11-167

**Published:** 2013-07-09

**Authors:** Mohit K Verma, Yasuhiro Miki, Keiko Abe, Takashi Suzuki, Hiromichi Niikawa, Satoshi Suzuki, Takashi Kondo, Hironobu Sasano

**Affiliations:** 1 Department of Pathology, Tohoku University Graduate School of Medicine, Sendai, Japan; 2 Department of Dentistry, Tohoku University Graduate School of Dentistry, Sendai, Japan; 3 Department of Pathology and Histotechnology, Tohoku University Graduate School of Health Sciences, Sendai, Japan; 4 Department of Thoracic Surgery, Institute of Development, Aging and Cancer, Tohoku University, Sendai, Japan; 5 Department of Thoracic Surgery, Ishinomaki Red Cross hospital, Ishinomaki, Japan; 6 Present address: The Jackson Laboratory, Bar Harbor, ME 04609 USA

**Keywords:** Lung cancer, Intratumoral estrogens, 17β-hydroxysteroid dehydrogenase, Targeted therapy

## Abstract

**Background:**

Estrogens were recently demonstrated to be synthesized in non-small cell lung carcinomas (NSCLCs) *via* aromatase activity and aromatase inhibitor (AI) did suppressed estrogen receptor (ER) positive NSCLC growth. However, other enzymes involved in intratumoral production and metabolism of estrogens, i.e. 17β-hydroxysteroid dehydrogenases (*i*.*e*. 17βHSD1 and 17βHSD2) and others have not been studied. Therefore, in this study, we examined the clinical/ biological significance of 17β-hydroxysteroid dehydrogenases in NSCLCs.

**Methodology:**

Archival materials obtained from 103 NSCLC patients were immunohistochemically evaluated using anti-17βHSD1 and anti-17βHSD2 antibodies. The findings of immunohistochemistry were then correlated with intratumoral estrone (E1) and estradiol (E2) concentration, clinicopathological factors and overall survival of the patients. We further employed NSCLC cell lines, A549 and LK87 to study the functional significance of 17βHSD1, *in vitro*.

**Results:**

A higher 17βHSD1 immunoreactivity tended to be positively associated with aromatase (*p*=0.057) and tumor stage (*p*=0.055) whereas a higher 17βHSD2 immunoreactivity was positively associated with a squamous cell and adenosquamous cell carcinomas subtypes (*p*=0.031), tumor stage (p=0.004), T factor of TNM classification (*p*=0.010), maximum tumor diameter (*p*=0.002) and tended to be associated with N factor of TMN classification (*p*=0.065). A higher 17βHSD1 immunoreactivity was also significantly associated with lower intratumoral E1 concentration (p=0.040) and a higher intratumoral E2/E1 concentration ratio (p=0.028). On the other hand a higher 17βHSD2 immunoreactivity was significantly associated with higher intratumoral E1 concentration (p=0.035). Results of multivariate regression analysis demonstrated an increased 17βHSD1 immunoreactivity in tumor cells as an independent negative prognostic factor (HR= 2.83, *p*=0.007). E1 treatment in 17βHSD1 positive NSCLC cells, A549 and LK87, resulted in E2 production (*p*<0.0001) and enhanced cell proliferation, which was abrogated effectively by 17βHSD1 siRNA knockdown (*p*<0.0001). In addition, aromatase inhibitor treatment resulted in 17βHSD1 up regulation in both A549 and LK87 cells.

**Conclusion:**

Results of our present study suggest that 17βHSD1 may be considered an important prognostic factor in NSCLC patients and targeting 17βHSD1 activity may further improve the clinical response in estrogen responsive NSCLC patients.

## Introduction

Lung cancer is the leading cause of cancer mortality worldwide [1]. Tobacco smoking still remains its prime cause among both men and women [2] but recent studies have also demonstrated that estrogens may contribute to the cell proliferation of some non-small cell lung carcinoma (NSCLC) cells [3]. Estrogens exert their effects *via* estrogen receptors (ERs) which are reported to be expressed very frequently in human NSCLCs of either gender, especially ERβ [4]. Both genomic and non-genomic actions of estradiol (E2) via ERβ have been reported in NSCLC cells; which result in tumor progression [5]. Therefore, at least some NSCLC are reasonably considered novel estrogen dependent neoplasms.

Male NSCLC patients with a high free E2 serum levels had significantly worse clinical outcome compared to those with lower E2 levels [6]. However, a frequent aromatase expression [7] and the ability of local production of estrogens *via* aromatase in estrogen dependent lung carcinoma cells have also been reported [8]. Due to the frequent expression of aromatase in NSCLC patients a phase II randomized trial of aromatase inhibitor (anastrozole) and ER blocker (fulvestrant) as consolidation therapy in postmenopausal women with advanced NSCLC was scheduled [9]. However, it is important to note that aromatase is not the only estrogen producing enzyme and other enzymes involved with intratumoral production and metabolism of estrogens, i.e. 17β-hydroxysteroid dehydrogenases (*i*.*e*. 17βHSD1 and 17βHSD2), steroid sulphatse (STS), estrone sulfotransferase (EST) could also be involved in modulation of intratumoral estrogens levels in NSCLCs. However, there have been no studies published on possible roles of 17βHSDs, STS and EST in NSCLC patients.

17βHSD1 catalyzes primarily the reduction of estrone (E1) to E2, whereas 17βHSD2 catalyzes primarily the oxidation of E2 to E1, and together they modulate the overall tissue levels of bioactive E2 in peripheral tissues including breast cancer [10]. An inhibitory potential of various novel 17βHSD1 inhibitors have been demonstrated for the treatment of estrogen-dependent diseases [11-13].

Based upon the reported findings above, we tentatively hypothesized that 17βHSDs pathway may play important roles in lung tumor progression *via* intratumoral estrogens production and regulation. Therefore, in this study, we first evaluated the status of both 17βHSD1 and 17βHSD2 in 103 NSCLC patients using immunohistochemistry (IHC). We then studied the correlation of the findings with clinicopathological variables, intratumoral E1 and/or intratumoral E2 tissue concentrations and overall survival in individual patients. The activity and regulation of 17βHSD1 was further examined in NSCLC cell lines i.e. A549 and LK87.

## Materials and methods

### Patients

103 NSCLC cases were retrieved from surgical pathology files of Department of Pathology, Tohoku University Hospital who underwent surgery from 1993 to 2003. Neither anti-EGFR nor anti-hormonal therapy was administered to any of the patients examined prior to surgery. Informed consent was obtained from each patient before surgery. Research protocols for this study were approved by the Ethics Committee at Tohoku University School of Medicine (Approval No. 2009–500).

### Immunohistochemistry

Serial tissue sections of 3 μm thickness fixed in 10% formaldehyde solution and embedded in paraffin were used for both hematoxylin-eosin staining and immunohistochemistry using labeled streptavidin biotin method. The primary antibodies used in this study are given as Additional file 1[14]. Positive controls were invasive ductal carcinoma of the breast for ERα, adenocarcinoma of the prostate for ERβ, tonsil for Ki67 and human full term placenta for aromatase, 17βHSD1 and 17βHSD2. As a negative control, normal mouse or rabbit IgG was used instead of the primary antibodies and no specific immunoreactivity was detected in these sections (data not shown). Immunoreactivity of ERα, ERβ, Ki-67/MIB1 and steroidogenic enzymes i.e. aromatase, 17βHSD1 and 17βHSD2 was counted among 1000 cells per case at hot spots and was determined as “positive” if immunereactivity was detected in more than 10% of cells, as previously described [15-17]. Based on the relative immunointensity of 17βHSD1 and/or 17βHSD2 in cytoplasm of the patients, the cases were classified as low (negative or weakly positive) and high (moderately/strongly positive), also according to the previous report [18]. The evaluation of immunohistochemical stains was done independently by two of the authors (M.K.V. and T.S.) that were blinded to the results for each antibody.

### Liquid chromatography/electrospray tandem mass spectrometry

Among 103 NSCLC patients, 48 paired frozen specimen of lung carcinomas and corresponding non-neoplastic lung tissues were available for liquid chromatography/electrospray tandem mass spectrometry for measurement of intratumoral E1 concentrations as previously reported [17,19]. We previously reported intratumoral E2 concentrations in these 48 patients [19]. The detailed methods of analyzing the intratumoral estrogens concentrations were also described in the report above.

### Cell culture and chemicals

Human NSCLC cell lines i.e. A549 and LK87 were provided by Institute of Development, Aging and Cancer, Tohoku University. Both of the cell lines were lung adenocarcinomas of male origin i.e. A549 (ATCC data sheet) and LK87 [20,21]. The cells were cultured in RPMI 1640 (Sigma-Aldrich) with 10% fetal bovine serum (Nichirei Co. Ltd.). Cells were incubated at 37°C in a humidified atmosphere containing 5% CO_2_. E1, E2 and testosterone were commercially obtained from Sigma-Aldrich.

### Quantitative RT-PCR

Total RNA was extracted using TRIzol reagent (Invitrogen Life Technologies) and cDNA was synthesized using a QuantiTect reverse transcription kit (Qiagen). Quantitative real-time PCR was carried out using the LightCycler System and FastStart DNA Master SYBR Green I (Roche Diagnostics). Ribosomal protein L 13a (RPL13A) was also used as an internal standard. The primer sequences used in this study are given as Additional file 2.

### ELISA assay

Cells were serum starved for 24 hours in a 24-well plate and then treated with steroids for 24 hours. Supernatant media was collected and evaluated for estrogen production using Estradiol EIA Kit (Cayman Chemical Company) according to the manufacturer’s protocol. An appropriate standard curve was plotted and the concentration of estradiol was determined.

### siRNA transfections

One scramble siRNA as a control and two 17βHSD1 specific siRNA were purchased from Sigma-Aldrich. The sense and antisense sequences of the two 17β-HSD1 siRNA, as described in the previous report [22] are given as Additional file 3. 200 nM of either 17βHSD1 siRNA 1 or 2 was transfected in NSCLC cell lines, i.e. A549 and LK87 cells, using G-fectin kit (Genolution Pharmaceuticals, Inc.) according to manufacturer’s protocol. Cells were harvested after 72 hours of siRNA transfections and evaluated for 17βHSD1 expression and/or activity.

### Cell proliferation assay

Cells were serum starved for 24 hours in a 96-well plate and then treated with test compounds and/or siRNA transfections for 72 hours. Then cells were harvested and evaluated for cell proliferation using WST-8 method (Cell Counting Kit-8; DOJINDO Laboratories). Optical densities (OD, 450 nm) were obtained with a microplate spectrophotometry (Model680, Bio-Rad Laboratories).

### Immunoblotting

Cells were serum starved for 24 hours in a 6 well plate and then treated with test compounds and/or siRNA transfections for an adequate length of time. 10 micrograms of the proteins from the cells were subjected to SDS-PAGE (10% acrylamide gel, SuperSep™Ace: Wako). The primary antibodies used were anti-17βHSD1 (1:1000; clone 2E5; Abnova) and anti-β-actin (1:1000; clone AC-15, Sigma–Aldrich). These antibody–protein complexes on the blots were detected using ECL-plus Western blotting detection reagents (GE Healthcare) using LAS-1000 cooled CCD-camera chemiluminescent image analyzer (Fuji Photo Film Co., Ltd.).

### Statistical analysis

For the statistical analysis in this study, we used STATVIEW version 5.0 (J software, SAS Institute Inc., USA). Statistical analyses used Fisher’s PLSD test, Scheffe’s F test, Bonferroni/Dunn test, Kaplan-Meier test, Logrank (Mantel-Cox) test and Student’s *t* test. A *p*-value less than 0.05 were considered to be significant.

## Results

### Immunohistochemistry of 17βHSD1 and 17βHSD2 in NSCLC patients

**Figure 1:**
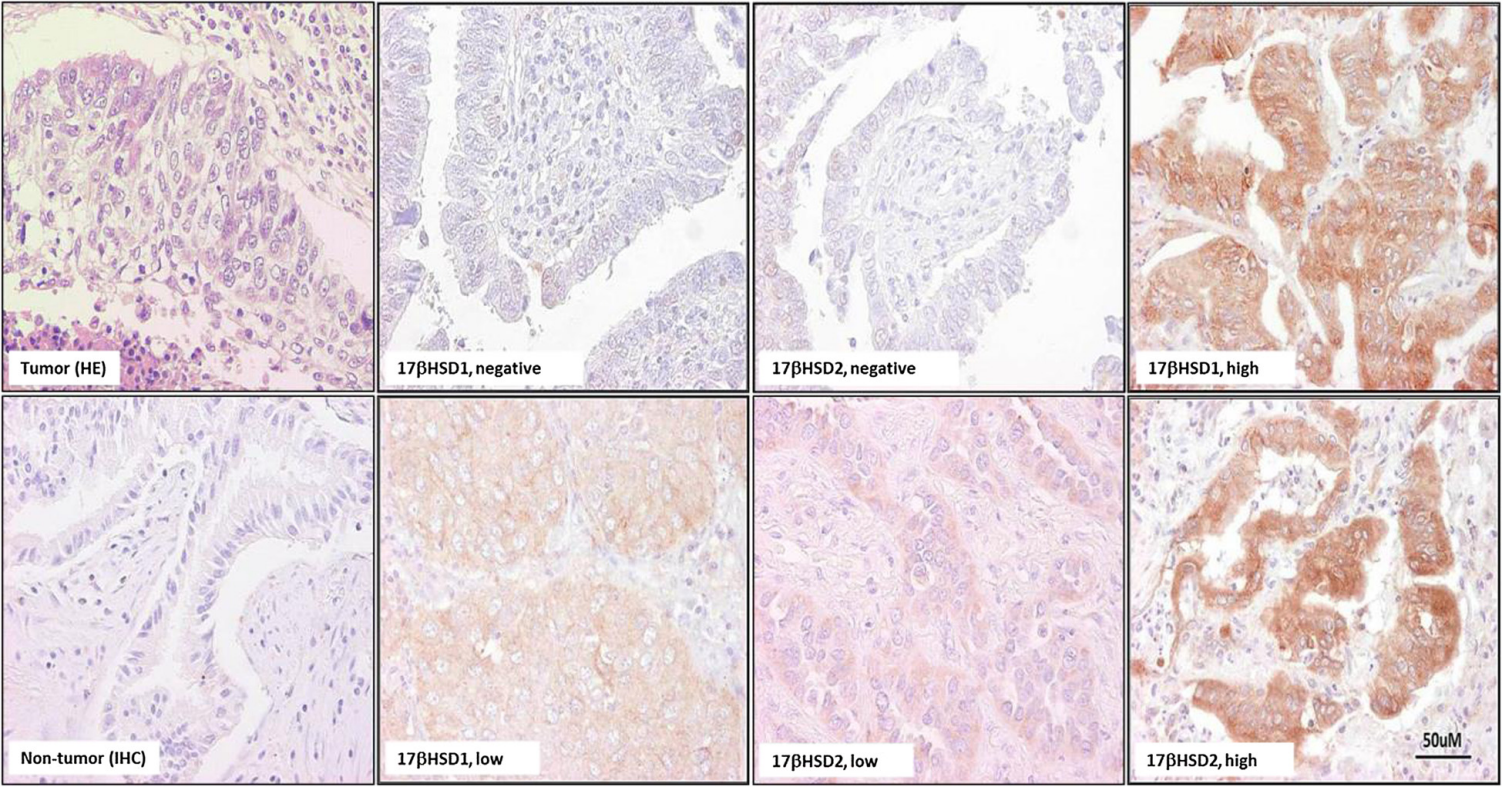
**Representative illustrations of immunohistochemistry of 17βHSD1 and 17βHSD2 in cases of NSCLCs.** Immunoreactivity was detected in the cytoplasm with varying intensity patterns, i.e. no staining, low staining and high staining.

Clinicopathological characteristics of the 103 NSCLC patients examined were summarized in Tables 1 and 2. 17βHSD1 and 17βHSD2 immunoreactivity was detected in the cytoplasm of 85% and 95% of the patients, respectively (Table 1, Figure 1). A high 17βHSD1 immunoreactivity tended to be positively associated with tumor stage (p=0.055) and aromatase (p=0.057), (Table 2). A high 17βHSD2 immunoreactivity status was positively associated with squamous cell and adenosquamous cell carcinomas subtypes (*p*=0.031), tumor stage (p=0.004), T factor of TNM classification (*p*=0.010), maximum tumor diameter (*p*=0.002) and tended to be associated with N factor of TMN classification (*p*=0.065) (Table 2). In addition, among 17βHSD1 and 17βHSD2 double positive patients (85 cases), a high status of 17βHSD1 was significantly associated with that of 17βHSD2 (*p*=0.014).

**Table 1:** Clinical and pathologic features of 103 NSCLC patients

**Clinicopathologic characteristics**	**No. of cases (%)**
**Age**	45-82 years
**Sex**	
Male	62 (60.2%)
Female	41 (39.8%)
**Histology**	
Adenocarcinoma	77 (74.7%)
Squamous cell carcinoma	23 (22.3%)
Adenosquamous cell carcinoma	3 (2.9%)
**Tumor size**	
T1	47 (45.6%)
T2	45 (43.7%)
T3	5 (4.8%)
T4	6 (5.8%)
**Lymph node metastases**	
N0	78 (75.7%)
N1	9 (8.7%)
N2	16 (15.5%)
**Distance metastasis**	
M0	99 (96.1%)
M1	4 (3.8%)
**17βHSDs**	
17βHSD1	88 (85.4%)
17βHSD2	98 (95.1%)
**ERα**	24 (23.3%)
**ERβ**	92 (89.3%)
**Aromatase**	86 (83.4%)

**Table 2:** Association between clinicopathological variables and 17βHSD1/ 17βHSD2 status in 103 NSCLC patients

**103 cases**		**17βHSD1**			**17βHSD2**		
		**High n=51**	**Low/Negative n=52**	**P**	**High n=54**	**Low/Negative n=49**	**P**
SEX	Male	31	31		36	26	
	Female	20	21	0.903	18	23	0.158
Histology	Adeno	38	39		35	42	
	SCC	11	12		16	7	
	Adeno squamous	2	1	0.826	3	0	**0.031**
Stage	I	28	40		27	41	
	II	7	2		7	2	
	III	15	8		18	5	
	IV	1	2	**0.055**	2	1	**0.004**
pT	pT1	20	27		18	29	
	pT2	25	20		26	19	
	pT3	3	2		4	1	
	pT4	3	4	0.617	6	0	**0.010**
pN	pN0	34	44		36	42	
	pN1	6	3		7	2	
	pN2	11	5	**0.100**	11	5	**0.065**
pM	pM0	50	49		52	47	
	pM1	1	3	0.306	2	2	0.921
Diameter*		15-65 (30)	10-90 (27)	0.156	18-75 (31.5)	10-90 (24)	**0.002**
Ki-67 %*		0-54.2 (17.6)	0-47.2 (16.6)	0.697	0-54.2 (18.3)	0-47.2 (13.4)	0.127
ERα	positive	15	9	0.244	14	10	0.641
	negative	37	42		40	39	
ERβ	positive	49	43	**0.097**	50	42	0.257
	negative	3	8		4	7	
Aromatase	positive	47	39	**0.057**	47	39	0.309
	negative	5	12		7	10	

Statistical analysis was conducted by Fisher exact test, Wilcoxon rank sum test, and Pearson *χ*^*2*^ test.

* Data were continuous variables and the median with minimum-maximum values were presented.

### Association of 17βHSD1 and 17βHSD2 with intratumoral tissue Estrone and Estradiol concentrations in NSCLC patients

**Figure 2:**
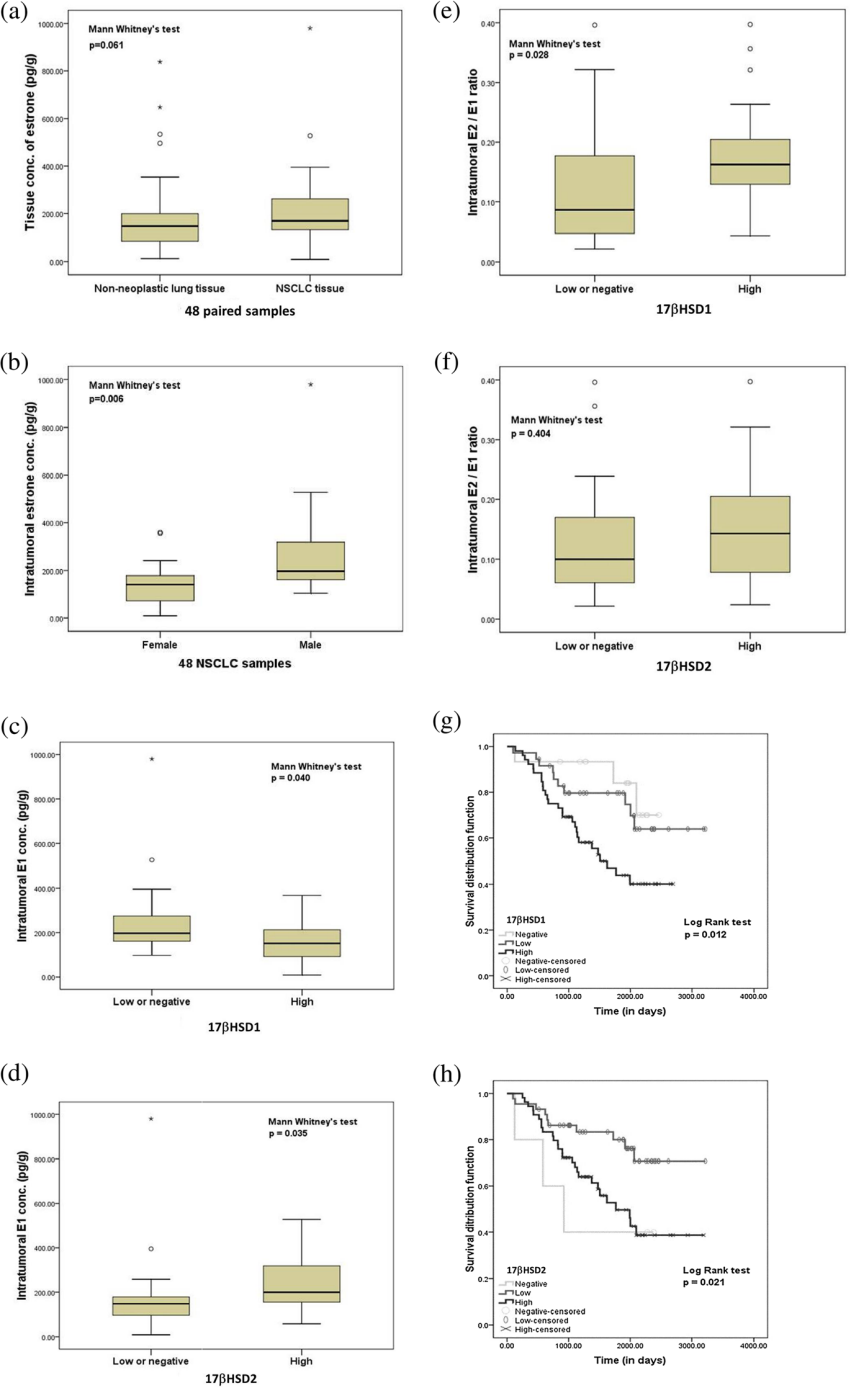
**Intratumoral estrogens concentration and overall survival in NSCLC patients. (a)** Intratumoral concentration of estrone in 48 NSCLCs and corresponding non-neoplastic lung tissues. **(b)** Intratumoral concentration of E1 in male and female NSCLCs. Association between intratumoral E1 concentration in 49 NSCLCs, **(c)** either with 17βHSD1 or **(d)** with 17βHSD2 enzymes. Association between intratumoral E2/E1 concentration in 49 NSCLCs, **(e)** either with 17βHSD1 or **(f)** with 17βHSD2 enzymes. The grouped data are represented as box plots. The median value is shown by a horizontal line in the box plot and the box denotes the 75th (upper margin) and 25th percentiles of the values (lower margin). Kaplan–Meier survival curves in 103 NSCLC patients, **(g)** according to 17βHSD1 immunoreactivity; negative vs. low vs. high and **(h)** according to 17βHSD2 immunoreactivity; negative vs. low vs. high.

The median with minimum-maximum value of tissue concentration of E1 and E2 in lung cancer tissues was 170.5 pg/g (9.2-979.88) and 20.35 pg/g (4.2-233.88) in this study. Thirty-four (71%) of 48 NSCLC cases demonstrated higher E1 tissue concentration in carcinoma tissues than the corresponding non-neoplastic lung tissues from the same patients, and these intratumoral E1 concentrations tended to be higher than those detected in their corresponding non-neoplastic lung tissues [148.6 (12.7-837.87) pg/g], (*p*=0.061), (Figure 2a). Tissue concentrations of E1 in men were significantly higher than those detected in postmenopausal women both in NSCLC (199.38 (104.43-979.88) pg/g in men and 141.11 (9.2-359.5) pg/g in postmenopausal women, *p*=0.006), (Figure 2b) and non-neoplastic lung tissues (173.59 (72.44-837.87) pg/g in men and 85.68 (12.7-281.2) pg/g in postmenopausal women, *p*<0.0001). Tissue E1 concentrations were significantly lower in NSCLC with a high 17βHSD1 status than that in patients with a low/negative 17βHSD1 status (151.57 (9.2-367.25) pg/g in high 17βHSD1 status and 197.08 (97.5-979.88) pg/g in low/negative 17βHSD1 status, *p*=0.040) (Figure 2c). Tissue E1 concentrations were, however, significantly higher in NSCLC with a high 17βHSD2 status than that in patients with a low or negative 17βHSD2 status (194.28 (58.64-527.7) pg/g in high 17βHSD2 status and 163.89 (9.2-979.88) pg/g in low/negative 17βHSD2 status, *p*=0.035) (Figure. 2(d)). There were no significant differences in intratumoral E2 concentration according to 17βHSD1 status (*p*=0.613) and/or 17βHSD2 status (*p*=0.177) (data not shown) but a higher intratumoral E2/ E1 ratio was significantly associated with a high 17βHSD1 status than that in those with a low/negative 17βHSD1 status (*p*=0.028) (Figure 2e). No significant differences were detected in intratumoral E2/ E1 ratio between a high 17βHSD2 status and that in patients with a low/negative 17βHSD1 status (*p*=0.404), (Figure 2f).

### Association of 17βHSD1 and 17βHSD2 with clinical outcome of NSCLC patients

17βHSD1 negative cases were significantly associated with better overall survival when compared to 17βHSD1 positive ones (*p*=0.012), (Figure 2g). 17βHSD2 negative cases were significantly associated with poorer overall survival when compared to 17βHSD2 positive patients (*p*=0.021), (Figure 2h) but cases with both higher 17βHSD1 and 17βHSD2 status when compared to those with both low status were positively associated with worse overall survival in NSCLC patient (*p*=0.012) and (*p*=0.021) respectively (Figure 2g,h). A subsequent multivariate regression analysis, however, demonstrated that only high 17βHSD1 status (HR= 2.83, *p*=0.007), sex (HR= 2.22, *p*=0.039), tumor stage (HR= 3.97, *p*=0.041), pT status (*p*=0.042), (HR= 2.83, *p*=0.007) and maximum tumor diameter (HR=6.53, *p*=0.001) turned out independent prognostic factors (Table 3).

**Table 3:** Univariate and multivariate analyses for clinical outcome in 103 NSCLC patients

**Variables**	**Overall survival**		**Hazard ratio**
	**Univariate**	**Multivariate**	**(95% CI)**
SEX	0.010*	**0.039**	**2.22 (.90-5.49)**
Histology	0.508	-	-
Stage (I + II vs. III +IV)	<0.0001*	**0.041**	**3.97 (1.05-15.02)**
pT	<0.0001*	**0.042**	-
pN	<0.0001*	0.054	-
pM	0.585	-	-
Diameter (≥30 mm vs. <30mm)	0.002*	**0.001**	**6.53 (2.14-19.86)**
Ki-67 labeling (≥10% vs. < 10%)	0.010*	0.284	1.76 (0.62-4.98)
17βHSD1 (high vs. low/negative)	0.003*	**0.007**	**2.83 (1.31-6.09)**
17βHSD2 (high vs. low/negative)	0.023*	0.286	0.62 (0.26-1.48)
ERα expression	0.599	-	-
ERβ expression	0.570	-	-
Aromatase expression	0.639	-	-

*Data were considered significant in the univariate analyses, and were examined in the multivariate analyses.

### Expression of 17βHSD1 and 17βHSD2 in NSCLC cell lines

**Figure 3:**
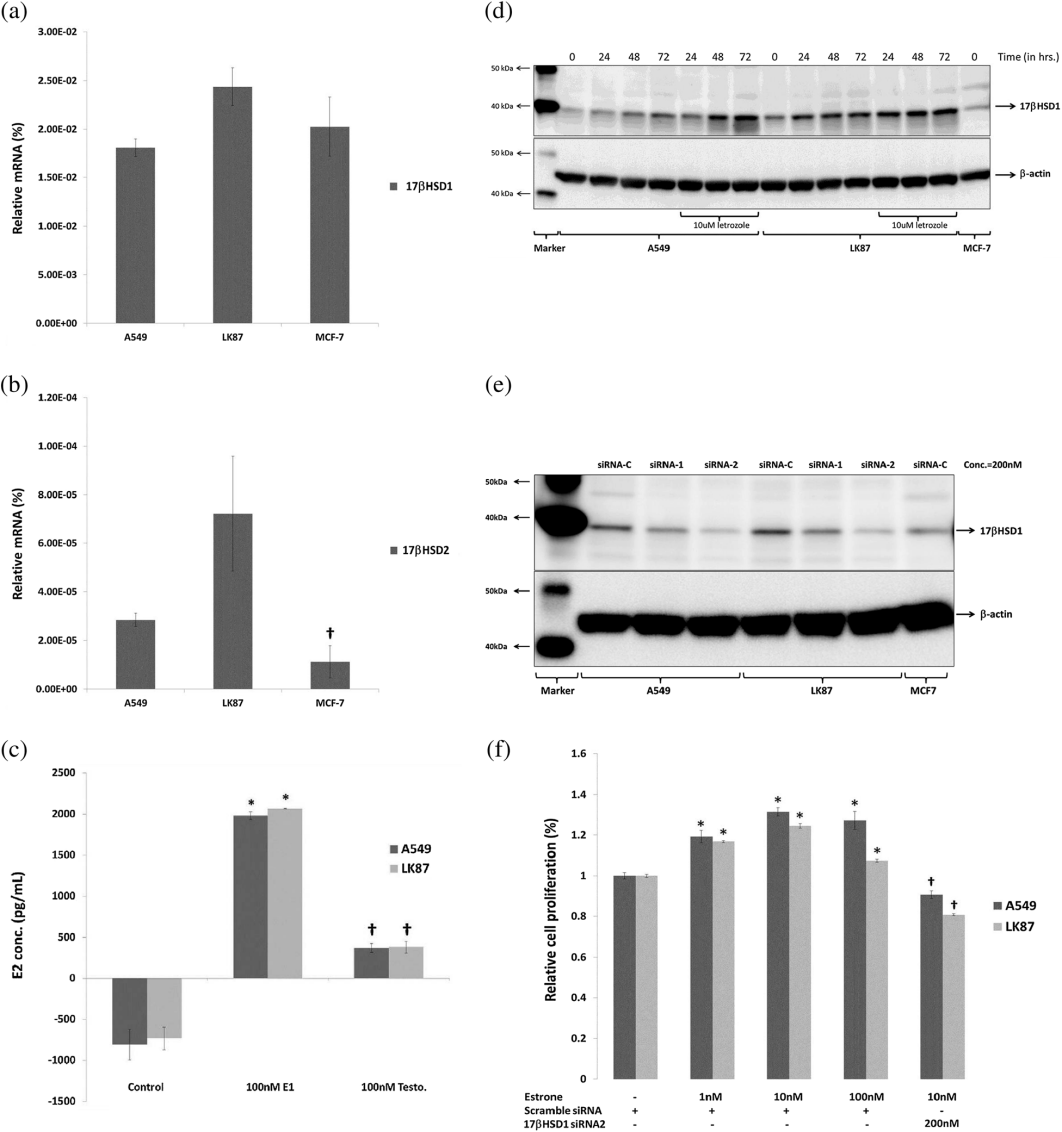
**17βHSD1 expression and activity in NSCLC cell lines. (a)** mRNA expression of 17βHSD1 and **(b)** 17βHSD2 enzymes. Positive control, MCF-7 cells. **(c)** E2 production via intratumoral 17βHSD1 activity and intratumoral aromatase activity on 24hrs of treatment with either E1 or testosterone, respectively. **(d)** Expression of 17βHSD1 following aromatase inhibitor treatment. **(e)** 17βHSD1 siRNA assisted 17βHSD1 knockdown after 72hrs of siRNA treatment. siRNA-C: scramble siRNA; siRNA1: 17βHSD1 specific siRNA1 and siRNA2: 17βHSD1 specific siRNA2. **(f)** Proliferation after 72hrs of treatment with either with E1 and/or 17βHSD1 specific siRNA. Data represent 3 independent experiments, each performed in triplicate. Results are represented as mean ± SD. All immunoblots are representative of 3 independent experiments.

We employed NSCLC cell lines A549 and LK87 expressing both aromatase and ERβ [23]. Quantitative RT-PCR analysis demonstrated that both A549 and LK87 cells express 17βHSD1 and 17βHSD2 mRNA (Figure 3a,b). The amounts of 17βHSD1 mRNA expression was significantly higher than that of 17βHSD2 in both of the NSCLC cell lines examined (*p*<0.0001). However, the amounts of 17βHSD2 mRNA expression was significantly higher in NSCLC cells than in breast carcinoma cells i.e. MCF-7, (*p*<0.0001).

### Targeting 17βHSD1 activity in NSCLC cells

A 24-hour treatment with 100nM E1 resulted in a significantly higher E2 production (5-fold) when compared to treatment with 100 nM testosterone in both A549 and LK87 cells (*p*=<0.0001), (Figure 3c). In addition, a 72 hour treatment with aromatase inhibitor (letrozole) resulted in up regulation of 17βHSD1 expression (Figure 3d). Two 17βHSD1 specific siRNA and a negative control of scramble siRNA were selected and used according to a previous report [22] in order to effectively knock down the expression of 17βHSD1 in these NSCLC cells. A 72 hour treatment with 17βHSD1 siRNA 2 significantly lowered endogenous 17βHSD1 expression in both A549 and LK87 cells (Figure 3e). In addition, a 72 hour E1 treatment increased cell proliferation in both of the NSCLC cell lines examined (*p*=<0.0001), which was also significantly inhibited by 17βHSD1 siRNA 2 transfection, (*p*=<0.0001) (Figure 3f).

## Discussion

In this study, we performed IHC analysis in 103 NSCLCs for evaluating the expression of 17βHSD1 and 17βHSD2 enzymes in NSCLC tissues. Both 17βHSD1 and 17βHSD2 were immunolocalized in the cytoplasm of carcinoma cells (Figure 1 Normal bronchial epithelial cells detected in the specimens were only occasionally weakly positive for either 17βHSD1 and/or 17βHSD2, suggesting a possible up-regulation of these enzymes in carcinoma cells. The majority of NSCLC cases examined in this study were positive for either 17βHSD1 (85%) and/or 17βHSD2 (95%) (Table 1). A relatively high number of NSCLC cases examined also demonstrated aromatase immunoreactivity (Table 1) as reported in many previous studies [7]. Intratumoral expression of estrogen producing enzymes i.e. aromatase, 17βHSD1 and 17βHSD2, all indicated that biologically active estrogens are actively synthesized either via aromatase and/or 17βHSDs pathway in NSCLC patients.

It is also true that 17βHSD1 and 17βHSD2 immunoreactivity was very heterogeneous in NSCLC cases (Figure 1). Therefore, based on relative immunointensity of either 17βHSD1 and/or 2 in cytoplasm of the tumor cells, the cases were tentatively sub-classified as low (negative or weakly positive) and high (moderately/strongly positive) as previously reported in breast cancer [18]. In breast cancer cases, the status of 17βHSD1 immunoreactivity in carcinoma cells was reported to be significantly correlated with that of ER and PgR, suggesting that E2, synthesized by 17βHSD1 in carcinoma cells may act on these cells locally [18]. However, in our present study of lung cancer, a relatively high immunoreactivity status of 17βHSD1 tended to be positively associated with ERβ (*p*=0.097), aromatase (*p*=0.057) and tumor stage (*p*=0.055) (Table 2), although that of 17βHSD2 was not associated with either ERβ (*p*=0.257) and/or aromatase status (*p*=0.309) (Table 2). In addition, the status of ERα immunoreactivity was by no means associated with either 17βHSD1 and/or 17βHSD2 statuses (Table 2), suggesting a more pronounced association between ERβ and 17βHSD pathway. These findings also suggest a potential interaction between intratumoral aromatase and 17βHSD pathways signaling in lung carcinoma cells. However, a high 17βHSD2 status was significantly associated with histological grade (*p*=0.031), tumor stage (*p*=0.004), T factor of TMN classification (*p*=0.010), maximum tumor diameter; (*p*=0.002) and also tended to be associated with N factor of TMN classification (*p*=0.065), (Table 2). These results indicated that 17βHSD2 may also play an important role in regulating lung cancer biology.

It then becomes important to evaluate the contribution of 17βHSD pathway in NSCLC patient towards an availability of intratumoral estrogens i.e. E2 and E1. In this study, intratumoral E1 was significantly higher in lung carcinoma tissue when compared to non-neoplastic tissue of the same NSCLC patients (Figure 2a). In addition, a high immmunointensity status of 17βHSD1 was associated with lower intratumoral E1 concentration and that of 17βHSD2 with higher intratumral E1 concentrations (Figure 2c,d). There were, however, no significant differences in intratumoral E2 concentration among patients having either high or low immmunointensity status of 17βHSD1 or 2 (data not shown). These results indicated that both 17βHSD1 and 17βHSD2 maintained a constant intratumoral E2 concentration equilibrium state by making use of available E2 and E1 *in situ*. Therefore, it is important to analyze whether these enzymes; i.e. 17βHSD1 and 17βHSD2, contribute to the intratumoarl E2/E1 ratio in NSCLC cells or not. A high E2/E1 ratio was reported to be positively correlated with the proliferation of estrogen dependent breast carcinoma cells and its reduction is also considered to be an effective mean of facilitating breast cancer therapy [22]. In this study, a higher intratumoral E2/E1 ratio was significantly associated with a high immmunoreactivity status of 17βHSD1 than that detected in patients with a low or negative 17βHSD1 status (p=0.028), (Figure 2e). These results demonstrated that intratumoral 17βHSD1 is also important for determining the E2/E1 ratio in lung carcinoma cells as in breast cancer patients and targeting 17βHSD1 could possibly confer therapeutic benefits to these estrogen dependent lung carcinoma patients.

In this study, among 17βHSD1 and 17βHSD2 double positive cases (85 cases), both 17βHSD1 and 17βHSD2 were significantly correlated with each other (*p*=0.014). In addition, the absence of 17βHSD1 in tumor cells was significantly associated with better overall survival (*p*=0.012) (Figure 2g) whereas the absence of 17βHSD2 with poorer overall survival of the patients examined (*p*=0.021), (Figure 2h). A high immmunoereactivity status of either 17βHSD1 (*p*=0.012) and/or 17βHSD2 (*p*=0.021) was also significantly associated with poorer overall survival when compared to lower statuses (Figure 2g,h). E2 is well-known to stimulate breast and endometrial carcinoma development. However, high doses of E2 are also well known to induce regression of hormone-dependent breast cancer in postmenopausal women and cause apoptosis of carcinoma cells [23]. Therefore, 17βHSD2 in NSCLC patients may play a role in protecting lung carcinoma cells from excessive stimulation of E2 by inactivation of potent E2 to inactive E1 and maintaining equilibrium status of constant E2 supply. Therefore, both 17βHSD1 and 17βHSD2 may work in tandem in order to maintain an equilibrium state of constant E2 supply in NSCLC tumor microenvironment. A multivariate regression analyses further demonstrated that only high 17βHSD1 status (HR= 2.83, *p*=0.007) and not high 17βHSD2 (*p*=0.286) status was an independent prognostic factor (Table 3). These data clearly demonstrated the prognostic significance of 17βHSD pathways in estrogen dependent NSCLC patients. In addition, in this study clinical impact of 17βHSD1 was much higher than that of ERβ (Table 3) suggesting that E2 may also have some other important functions besides ERβ mediated actions in NSCLCs, as suggested by earlier studies in breast cancer [24]. However, further investigations are definitely required for better clarifications.

Earlier studies demonstrated both A549 and LK87 cells as estrogen dependent NSCLC cell lines expressing aromatase [25,26]. Both of these cell lines expressed higher 17βHSD1 mRNA level when compared to 17βHSD2 suggesting a more pronounced role of 17βHSD1 in these NSCLC cells (Figure 3a,b). This 17βHSD1 activity resulted in significantly higher E2 production (5-fold) when compared to aromatase activity in both A549 and LK87 cells (*p*<0.0001) (Figure 3c). This difference could be due to a higher 17βHSD1 level when compared to aromatase in these NSCLC cell lines [25]. We observed an increment of both 17βHSD1 following AI therapy in these NSCLC cell lines suggesting a possible emergence of 17βHSD pathway, (Figure 3d). This increment may represent a possible adaptation of carcinoma cells in response to intratumoral estrogen depletion, in lung cancer cells. We have recently demonstrated a similar increment of 17βHSD1 following aromatase inhibitor (exemestane) treatment in the group associated with decreased Ki67 labeling index or clinical responders in breast cancer patients [27]. This result also suggests that 17βHSD enzymes may represent as a potent marker for response to aromatase inhibitor therapy in the lung carcinoma, but it awaits further examinations. Finally, E1 treatment stimulated cell proliferation in both cell lines above which was significantly inhibited by 17βHSD1 siRNA 2 transfection, (*p*<0.0001) (Figure 3f).

## Conclusion

These results all suggested that in addition to intratumoral production via aromatase pathway, 17βHSD1 is active and contributes to tumor progression of estrogen dependent NSCLC cells by making use of available intratumoral E1. Therefore, potential endocrine therapy targeting 17βHSD1 may translate into therapeutic benefits to estrogen dependent NSCLC patients. Therefore, similar to estrogen dependent breast cancer patients, 17βHSD1 inhibitors either alone or in combination with aromatase inhibitors may represent a novel approach for treating estrogen dependent lung cancer patients. It is also important to note that, in addition to 17βHSD1 and 17βHSD2, other sub-types of 17βHSDs were also reported to be implicated in various hormone dependent carcinomas [28]. These 17βHSD subtypes may therefore, be reasonably be postulated to play important roles in hormone dependent human lung cancer patients. However, further investigations are definitely required for clarifications.

## Competing interests

The authors declare that they have no competing interests.

## Authors’ contributions

MKV designed the study and wrote the manuscript. SS and TK collected the samples and provided clinical data. MKV and TS evaluated the immunohistochemical stains. HN contributed the intratumoral E2 concentration data. MKV performed the IHC, LC-MS, Real time PCR analysis, ELISA assay and siRNA transfections, Cell proliferation assay and Immunoblotting. MKV statistically analyzed and interpreted the data. YM, KA and HS contributed to the writing of the manuscript. All authors read and approved the final manuscript.

## Supplementary Material

Additional file 1 **The primary antibodies used in this study were as follows: (a)** anti-ERβ (1:50; clone 14C8; GeneTex). **(b) **anti-aromatase (1:3000; clone #677/H7, provided by Novartis) [16]. **(c)** anti-ERα?(1:50; clone 6F11; Novocastra). **(d)** anti-17βHSD1 (1:400; clone 2E5; Abnova). **(e)** anti-17βHSD2 (1:200; Proteintech). **(f)** anti-Ki-67 (1:100; clone MIB1, DakoCytomation). Click here for file

Additional file 2 **The primer sequences used in this study were as follows. (a) **17βHSD1: (NM_000413; forward: 1,290-1,310 and reverse: 1,604-1,623). **(b)**17βHSD2: (NM_002153; forward: 797–816 and reverse: 971–989). **(c) **RPL13A : (NM_012423; forward: 487–509 and reverse: 588–612). Click here for file

Additional file 3 **The sense and antisense sequences of the two 17βHSD1 siRNAs used in this study were as follows. (a)**17βHSD1 siRNA 1: Sense sequence (5’-3’); GCCUUUCAAUGACGUUUAU [dT][dT], Anti-sense sequence (3’-5’); AUAAACGUCAUUGAAAGGC [dT][dT]. **(b)**17βHSD1 siRNA 2: Sense sequence (5’-3’); CCACAGCAAGCAAGUCUUU [dT][dT], Anti-sense sequence (3’-5’); AAAGCAUUGCUUGCUGUGG [dT][dT]. Click here for file
