# Supplementary material for: Intratumoral localization and activity of 17β-hydroxysteroid dehydrogenase type 1 in non-small cell lung cancer: a potent prognostic factor
Source: J Transl Med. 2013 Jul 9;11:167. doi: 10.1186/1479-5876-11-167 (PMC3724709; doi:10.1186/1479-5876-11-167)
Supplement: Additional file 1 — The primary antibodies used in this study were as follows: (a) anti-ERβ (1:50; clone 14C8; GeneTex). (b) anti-aromatase (1:3000; clone #677/H7, provided by Novartis) [16]. (c) anti-ERα?(1:50; clone 6F11; Novocastra). (d) anti-17βHSD1 (1:400; clone 2E5; Abnova). (e) anti-17βHSD2 (1:200; Proteintech). (f) anti-Ki-67 (1:100; clone MIB1, DakoCytomation). [file 1479-5876-11-167-S1.doc]

**Additional file 1:**

1. The primary antibodies used in this study were as follows:
2. anti-ERβ (1:50; clone 14C8; GeneTex)
3. anti-aromatase (1:3000; clone #677/H7, provided by Novartis) [16]
4. anti-ERα (1:50; clone 6F11; Novocastra)
5. anti-17βHSD1 (1:400; clone 2E5; Abnova)
6. anti-17βHSD2 (1:200; Proteintech)
7. anti-Ki-67 (1:100; clone MIB1, DakoCytomation)
