# Supplementary material for: Intratumoral localization and activity of 17β-hydroxysteroid dehydrogenase type 1 in non-small cell lung cancer: a potent prognostic factor
Source: J Transl Med. 2013 Jul 9;11:167. doi: 10.1186/1479-5876-11-167 (PMC3724709; doi:10.1186/1479-5876-11-167)
Supplement: Additional file 2 — The primer sequences used in this study were as follows. (a) 17βHSD1: (NM_000413; forward: 1,290-1,310 and reverse: 1,604-1,623). (b)17βHSD2: (NM_002153; forward: 797–816 and reverse: 971–989). (c) RPL13A : (NM_012423; forward: 487–509 and reverse: 588–612). [file 1479-5876-11-167-S2.doc]

**Additional file 2:**

1. The primer sequences used in this study were as follows:
2. 17βHSD1: (NM_000413; forward: 1,290-1,310 and reverse: 1,604-1,623)
3. 17βHSD2: (NM_002153; forward: 797-816 and reverse: 971-989)
4. RPL13A : (NM_012423; forward: 487-509 and reverse: 588-612)
