# Supplementary material for: Intratumoral localization and activity of 17β-hydroxysteroid dehydrogenase type 1 in non-small cell lung cancer: a potent prognostic factor
Source: J Transl Med. 2013 Jul 9;11:167. doi: 10.1186/1479-5876-11-167 (PMC3724709; doi:10.1186/1479-5876-11-167)
Supplement: Additional file 3 — The sense and antisense sequences of the two 17βHSD1 siRNAs used in this study were as follows. (a)17βHSD1 siRNA 1: Sense sequence (5’-3’); GCCUUUCAAUGACGUUUAU [dT][dT], Anti-sense sequence (3’-5’); AUAAACGUCAUUGAAAGGC [dT][dT]. (b)17βHSD1 siRNA 2: Sense sequence (5’-3’); CCACAGCAAGCAAGUCUUU [dT][dT], Anti-sense sequence (3’-5’); AAAGCAUUGCUUGCUGUGG [dT][dT]. [file 1479-5876-11-167-S3.doc]

**Additional file 3:**

1. The sense and antisense sequences of the two 17βHSD1 siRNAs used in this study were as follows:
2. 17βHSD1 siRNA 1: Sense sequence (5’-3’); GCCUUUCAAUGACGUUUAU [dT][dT], Anti-sense sequence (3’-5’);　AUAAACGUCAUUGAAAGGC　[dT][dT]
3. 17βHSD1 siRNA 2: Sense sequence (5’-3’); CCACAGCAAGCAAGUCUUU [dT][dT], Anti-sense sequence (3’-5’); AAAGCAUUGCUUGCUGUGG [dT][dT].
